# A novel *de novo* heterozygous *DYRK1A* mutation causes complete loss of DYRK1A function and developmental delay

**DOI:** 10.1038/s41598-020-66750-y

**Published:** 2020-06-17

**Authors:** Kyu-Sun Lee, Miri Choi, Dae-Woo Kwon, Doyoun Kim, Jong-Moon Choi, Ae-Kyeong Kim, Youngwook Ham, Sang-Bae Han, Sungchan Cho, Chong Kun Cheon

**Affiliations:** 1 Bionanotechnology Research Center, Korea Research Institute of Bioscience and Biotechnology, 125 Gwahak-ro, Yuseong-gu, Daejeon, 34141 Republic of Korea; 2 Department of Functional Genomics, KRIBB School of Bioscience, Korea University of Science and Technology, 217 Gajeong-ro, Gajeong-dong, Yuseong-gu, Daejeon, 34113 Republic of Korea; 3 Natural Medicine Research Center, Korea Research Institute of Bioscience and Biotechnology, 30 Yeongudanji-ro, Ochang-eup, Cheongwon-gu, Cheongju-si, Chungbuk, 28116 Republic of Korea; 4 College of Pharmacy, Chungbuk National University, 30-1 Yeonje-ri, Osong-eup, Heungduk-gu, Cheongju-si, Chungbuk, 28644 Republic of Korea; 5 Innovative Target Research Center, Korea Research Institute of Chemical Technology, 141 Gajeong-ro, Jang-dong, Yuseong-gu, Daejeon, 34114 Republic of Korea; 6 Green Cross Genome, Green Cross Laboratories, 107 Ihyeon-ro 30 beon-gil, Giheung-gu, Yongin-si, Gyeonggi, 16924 Republic of Korea; 7 Department of Biomolecular Science, KRIBB School of Bioscience, Korea University of Science and Technology, 217 Gajeong-ro, Gajeong-dong, Yuseong-gu, Daejeon, 34113 Republic of Korea; 8 Division of Medical Genetics and Metabolism, Department of Pediatrics, Pusan National University Children’s Hospital, Pusan National University School of Medicine, 20 Geumo-ro, Mulgeum-eup, Yangsan-si, Gyeongnam, 50612 Republic of Korea; 9 Research Institute for Convergence of Biomedical Science and Technology, Pusan National University Yangsan Hospital, 20 Geumo-ro, Mulgeum-eup, Yangsan-si, Gyeongnam, 50612 Republic of Korea

**Keywords:** Developmental biology, Genetics, Diseases, Molecular medicine, Pathogenesis

## Abstract

Dual-specificity tyrosine phosphorylation-regulated kinase 1 A (DYRK1A) is essential for human development, and *DYRK1A* haploinsufficiency is associated with a recognizable developmental syndrome and variable clinical features. Here, we present a patient with *DYRK1A* haploinsufficiency syndrome, including facial dysmorphism, delayed motor development, cardiovascular system defects, and brain atrophy. Exome sequencing identified a novel *de novo* heterozygous mutation of the human *DYRK1A* gene (c.1185dup), which generated a translational termination codon and resulted in a C-terminally truncated protein (DYRK1A-E396ter). To study the molecular effect of this truncation, we generated mammalian cell and *Drosophila* models that recapitulated the DYRK1A protein truncation. Analysis of the structure and deformation energy of the mutant protein predicted a reduction in protein stability. Experimentally, the mutant protein was efficiently degraded by the ubiquitin-dependent proteasome pathway and was barely detectable in mammalian cells. More importantly, the mutant kinase was intrinsically inactive and had little negative impact on the wild-type protein. Similarly, the mutant protein had a minimal effect on *Drosophila* phenotypes, confirming its loss-of-function *in vivo*. Together, our results suggest that the novel heterozygous mutation of *DYRK1A* resulted in loss-of-function of the kinase activity of DYRK1A and may contribute to the developmental delay observed in the patient.

## Introduction

Dual-specificity tyrosine phosphorylation-regulated kinase 1A (DYRK1A) is a member of the highly conserved DYRK kinase family that belongs to the CMGC kinase superfamily. DYRK1A phosphorylates serine and threonine residues on its target substrates and autophosphorylates its own tyrosine residues^1,2^. Due to the variety of proteins that DYRK1A phosphorylates, it plays essential roles in a wide range of cellular signalling pathways and processes, including neurogenesis, neuronal differentiation and proliferation^3^, synaptic transmission^4^, cell cycle^5^, apoptosis^6^, splicing^7^, and RNA transcription^8^. Many of these pathways and processes regulate neurological function, and, indeed, DYRK1A overexpression or overactivity is associated with many neurodegenerative diseases, such as Alzheimer’s, Parkinson’s, Huntington’s, and Pick’s diseases, and even certain types of cancers (e.g., glioma)^9–14^. DYRK1A is also thought to contribute to several of the phenotypic and neurocognitive features of Down Syndrome (DS), because individuals with DS carry an extra copy of DYRK1A^15–17^. Decreased DYRK1A expression or activity is also pathogenic. For example, mice hemizygous for *DYRK1A* have substantial phenotypic defects, including smaller body size, microcephaly, reduced numbers of neurons, abnormal motor function, gait disturbances, and impaired cognitive function^18,19^. Human *DYRK1A* haploinsufficiency is generated by a variety of mutations and is a potential cause of a recognizable developmental syndrome that is characterized by variable clinical features, including intellectual disability, developmental delay, microcephaly, dysmorphic facial features, speech delay, autism, febrile seizures, and ocular malformations (OMIM: 614104, ORPHANET: 464306)^20,21^. Individuals with this syndrome were first identified with partial monosomies of chromosome 21 on routine karyotypes that encompassed the *DYRK1A* gene (21q22.13)^22^. More recently, the diagnosis of numerous *de novo* mutations in *DYRK1A* has been achieved by next generation sequencing, which has facilitated and broadened the clinical characterization of *DYRK1A* disruptions. To date, many mutations associated with *DYRK1A* have been identified and include gross deletions, small deletions, point mutations, complex rearrangements, small indels, and splice-site mutations (Human Gene Mutation Database, http://www.hgmd.org). Many of these mutations result in truncated proteins that partially or completely lack the DYRK1A kinase domain and thereby lose their catalytic activity.

Here, we report a novel *de novo DYRK1A* mutation occurring in the β-sheet of the CMGC insert, which is located in the C-terminal end of the kinase domain. This nonsense mutation led to the production of a C-terminally truncated kinase domain protein (DYRK1A-E396ter). The resulting mutant protein was not only efficiently degraded by the proteasome but was also catalytically inactive in mammalian cell and fly models, indicating complete loss-of-function of DYRK1A.

## Materials and Methods

### Patient

The study was approved by the Institutional Review Board of Pusan National University Yangsan Hospital (approval number: 05-2019-103) and adhered to the tenets of the Declaration of Helsinki involving ethical principles for medical research with human subjects. Informed consent was obtained from the child’s parents.

### Genetic analysis

Written informed consent was obtained from all participants before blood was drawn. Genomic DNA was isolated using the QIAamp DNA Blood Midi kit (Qiagen, Hilden, Germany) from participants’ leukocytes in the peripheral blood, according to the manufacturer’s standard protocols. The extracted gDNA was evaluated using the TruSight One Sequencing Panel (Illumina Inc., San Diego, CA, USA) as described previously^23^. Captured targeted regions were sequenced using the Hiseq 2500 Sequencing System (Illumina Inc.) following the manufacturer’s instructions. Alignment and variant calling was done automatically by on-instrument tools. Imported sequence data was filtered for specified genes and converted into a customized report using the VariantStudio software. Pathogenic variants were evaluated by the practical statement released by the American College of Medical Genetics and Genomics^24^.

### Plasmid construction

To construct plasmids expressing FLAG-DYRK1A proteins, the DNA fragment encoding FLAG (DYKDDDDK) was inserted into a pcDNA3.1(+) vector at *NheI/HindIII* sites, and the open reading frame of human *DYRK1A* (NM_001396.4) was cloned into a pcDNA3.1(+) vector at *HindIII/XbaI* sites. Plasmids expressing FLAG-DYRK1A-E396ter and FLAG-DYRK1A-K188R were generated by mutating the original sequence with a QuikChange II Site-Directed Mutagenesis Kit (Agilent Technologies, Santa Clara, CA, USA), according to the manufacturer’s method. The following primers that are specific to each mutant were used: 5ʹ-CAAAAGCAAGAAAGTTCTTTTGAGAAGTTGCCAGATG-3’ (forward) and 5ʹ-CATCTGGCAACTTCTCAAAAGAACTTTCTTGCTTTTG-3ʹ (reverse) for FLAG-DYRK1A-E396ter; 5ʹ-CAAGAATGGGTTGCCATTAGAATAATAAAGAACAAGAAG-3ʹ (forward) and 5ʹ-CTTCTTGTTCTTTATTATTCTAATGGCAACCCATTCTTG-3ʹ (reverse) for FLAG-DYRK1A-K188R.

### Cell culture and transfection

Human embryonic kidney 293T cells were cultured in Dulbecco’s Modified Eagle’s Medium containing 10% foetal bovine serum (Welgene, Gyeongsan-si, Gyeongsangbuk-do, Republic of Korea) supplemented with 1% streptomycin and penicillin. The cells were seeded at approximately 50% confluency into cell culture plates and were maintained overnight at 37 °C under 5% CO_2_. When the cells reached 60–80% confluency, they were transfected with plasmids using the XtremeGene Transfection Reagent (Roche, Basel, Switzerland), according to the manufacturer’s instructions. Transfected cells were incubated at 37 °C for 24 h prior to harvest or analysis.

### Chemicals

We used the proteasome inhibitor MG132 (Calbiochem, San Diego, CA, USA), the lysosomal inhibitor NH_4_Cl (Sigma-Aldrich, St. Louis, MO, USA), the calpain inhibitor calpeptin (Calbiochem), and the autophagy inhibitor 3-methyladenine (Sigma-Aldrich) for protein degradation pathway analyses. All chemicals were dissolved in dimethyl sulfoxide (DMSO) prior to treatment, and the cells were treated with 10 μM of each compound for 15 h after plasmid transfection.

### Quantitative western blot analysis

Quantitative western blot analysis was performed as described previously^25^. The following antibodies were used: anti-FLAG M2 antibody (catalogue #F1804; Sigma-Aldrich; 1:500 dilution), anti-HA antibody (catalogue #sc-805; Santa Cruz Biotechnology, Santa Cruz, CA, USA; 1:1,000 dilution), anti-hnRNP A1 antibody (catalogue #CSB-PA002942; Cusabio, Houston, TX, USA; 1:1,000 dilution), anti-Tau antibody (catalogue #MN1040; Thermo Fisher Scientific, Waltham, MA, USA; 1:1,000 dilution), and anti-phospho-Tau (at residue Thr-212) antibody (catalogue # 4–740 G; Thermo Fisher Scientific; 1:1,000 dilution). Signals were detected on a LAS-4000 image analyser using a Clarity^TM^ Western ECL Substrate (BIO-RAD, Hercules, CA, USA) and then quantitatively analysed using the NIH ImageJ software (National Institutes of Health, Bethesda, MD, USA).

### Quantitative RT-PCR (qRT-PCR)

The 293T cells were seeded in 6-well cell culture plates and transfected with FLAG-tagged wild-type or mutant DYRK1A plasmids for 24 h. TRIzol reagent (Invitrogen, Carlsbad, CA, USA) was used for total RNA extraction, and the Omniscript RT Kit (Qiagen, Hilden, Germany) was used to synthesize first-strand cDNA from 1 μg of total RNA, which was primed with oligo-dT primers. qRT-PCR was performed using the TOPreal SYBR Green PCR Kit (Enzynomics, Daejeon, Republic of Korea) in triplicate with the following primer sets: 5ʹ-TAGCATGGATTACAAGGATGACGATG-3ʹ (FLAG-DYRK1A forward), 5ʹ- ACATAAGTGACCAACAGGTTTCTGC-3ʹ (FLAG-DYRK1A reverse), 5ʹ-AGAGCTACGAGCTGCCTGAC-3ʹ (Human β-actin forward), and 5ʹ-AGCACTGTGTTGGCGTACAG-3ʹ (Human β-actin reverse).

### Polyubiquitination assay

Polyubiquitination assays were performed as described previously^26^. Briefly, the 293 T cells were co-transfected with FLAG-DYRK1A-E396ter and HA-ubiquitin plasmids using the XtremeGene Transfection Reagent (Roche) for 24 h. Cells were then treated with 10 μM MG132 for 11 h, harvested, and lysed in an immunoprecipitation (IP) buffer (Tris/HCl, pH 7.5, 150 mM NaCl, 2 mM EDTA and 1% NP-40). The cell lysates were mixed with anti-FLAG M2 antibody-conjugated protein A/G-agarose beads (Santa Cruz Biotechnology) in 0.2% NP-40-containing IP buffer using a tubing-rotator at 4 °C for 2 h. The bound beads were washed three times with 0.2% NP-40-containing IP buffer and were solubilized with 2X sample buffer for western blot analysis.

### ***Drosophila*culture**

*Drosophila melanogaster* were cultured at 25 °C on standard cornmeal media. *GMR-gal4* (#9146), *MS1096-gal4* (#8860), *Mhc-gal4* (#55133), *UAS-Tau* (human wild-type Tau, #51362)^27^, and all other stocks and balancers were obtained from the Bloomington Stock Centre (Bloomington, IN, USA). *UAS-mnb* transgenic flies were described in our previous report^28^. Based on amino acid sequence alignment, the human DYRK1A E396 residue corresponded to the *Drosophila* mnb D401 residue. Both wild-type full-length *mnb* and truncated mutant (*mnb-D401ter*) cDNAs were generated by PCR using *Drosophila* S2 cell cDNA as a template. Phusion Pfu PCR polymerase (Invitrogen) was used with the following primer pairs: 5ʹ-GAATTCATGTATAGATTAGAGGATACGA-3ʹ (forward, common for *mnb-WT* and *mnb-D401ter*), 5ʹ-TCTAGACTAATGTATAACTACAGGATTC-3ʹ (reverse for *mnb-WT*), and 5ʹ-TCTAGATCAGAAGAACTTGCGGGTCTTG-3ʹ (reverse for *mnb-D401ter*). The cDNAs of the full-length and truncated forms of *mnb* were ligated to the pUAST-FLAG vector digested with *EcoRI/XbaI* restriction enzymes to place the transgenes under the control of the UAS promoter. *UAS-mnb-WT* and *UAS-mnb-D401ter* transgenic flies were obtained by P-element-mediated germline transformation^29^. To analyse the eye phenotype, newly eclosed flies were collected and allowed to mate for 2–3 days. Wings from adult flies were dissected in 100% ethanol and mounted in Canada Balsam mounting medium (Gary’s magic mountant). The eye and wing phenotypes were photographed using a SZ60 binocular microscope equipped with an eXcope K5 CCD system (Olympus, Tokyo, Japan). Fly tissue sizes were measured on multiple samples (*n* > 10) from each genotype using NIH ImageJ software. The average eye size was presented as a normalized percentage of the control eye size. For adult lethality assessments, at least 100 flies were included in each individual experiment. For statistical analyses, two-tailed unpaired Student’s *t*-tests were used.

### Immunohistochemistry

Immunostaining of larval samples was performed as previously described^30^. Briefly, fixed larval preparations were washed in phosphate buffered saline with Tween-20 (PBST) three times (10 min each), blocked in PBST with 5% normal goat serum for 30 min, and incubated with primary antibody at room temperature for 2 h or at 4 °C overnight. Fluorescein isothiocyanate-labelled goat anti-horseradish peroxidase (1:50 dilution, The Jackson Laboratory, Bar Harbour, ME, USA) was used to detect the synaptic bouton in larval tissue. All images were collected using a FV1000 confocal microscope (Olympus) and processed using NIH ImageJ software. Neuromuscular junction (NMJ) quantifications were conducted based on published procedures^31^. The filopodial NMJ structure was processed from complete z-stacks using the entire NMJ of the A4 abdominal segment. All statistical comparisons were performed using Prism software (GraphPad, San Diego, CA, USA). *P-*values were calculated using two-tailed Student’s *t-*tests.

## Results

### Case presentation

**Figure 1:**
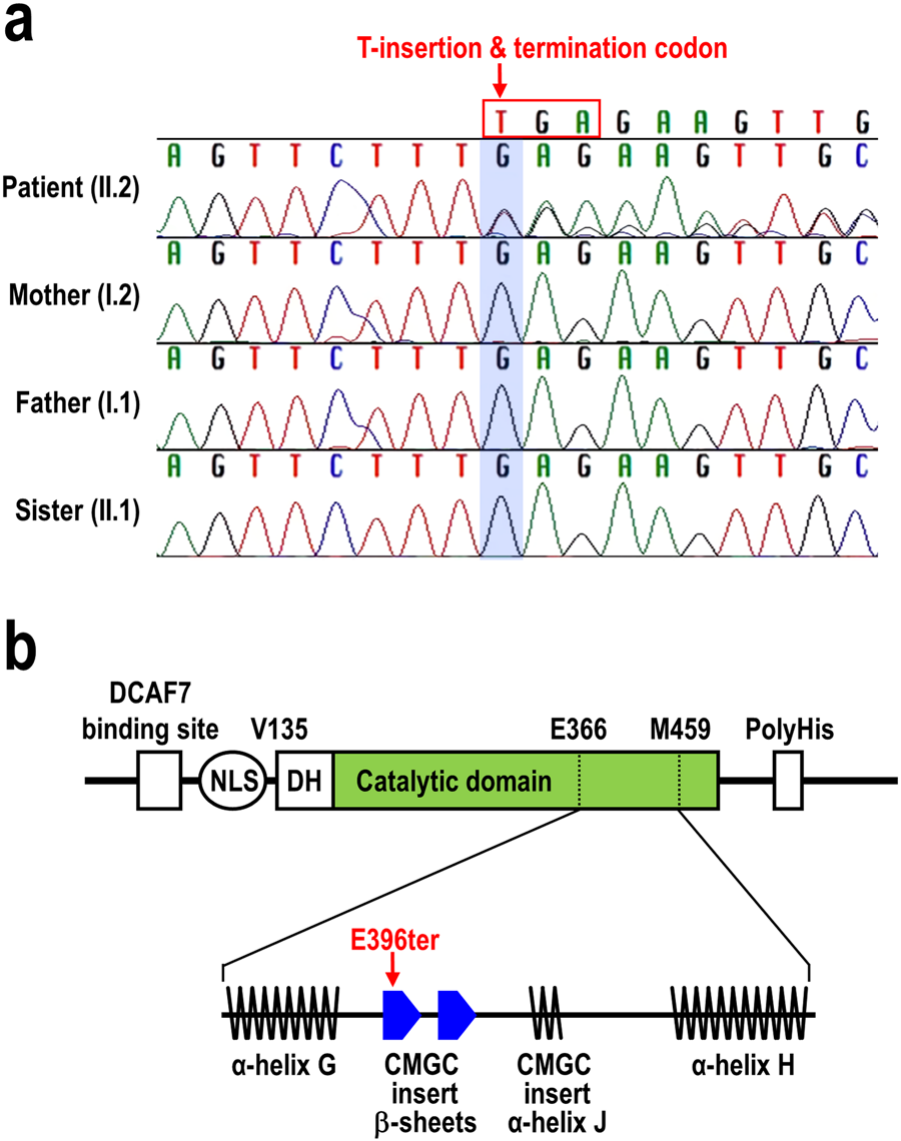
A novel *de novo* heterozygous mutation in the kinase domain of the *DYRK1A* gene. (**a**) Sanger sequencing confirmed a heterozygous novel *DYRK1A* variant, c.1185dup (p.E396ter) (NM_001396.3), in exon 8 that was identified by exome sequencing of the patient’s genome. (**b**) Schematic diagram of the DYRK1A catalytic domain. The location of the mutation and the translational termination codon (E396ter) is indicated as a red arrow.

A 5-month-old female was referred to our hospital for evaluation of delayed development and facial dysmorphism. The patient was born after 39 weeks and 4 days of gestation by vaginal delivery after an uneventful pregnancy and weighed 2.89 kg. She was the third child of healthy Korean parents (Supplementary Fig. S1), and her siblings showed normal development. Her phenotypic features included an epicanthal fold, tented mouth, short and deep philtrum, deep-set eyes, bi-temporal narrowing, micrognathia, wide nasal bone, sparse scalp hair, and prominent ears with underdeveloped ear lobes (Supplementary Fig. S1). She also displayed rigidity in both her upper and lower extremities as well as thumb folding and deep tendon reflexes. An echocardiogram showed that she had a secondary atrial septal defect and peripheral pulmonary stenosis. Her chromosomal microarray examination revealed normal results. According to the Bayley Test (BSID-III), which measures early development in children, the patient had a language delay (composite score: 56; percentile score: 0.2), motor delay (composite score: 46; percentile score: <0.1), and poor adaptive behaviour skills (composite score: 66; percentile score: 1). At the time of her visit to the hospital, approximately 5 months after birth, her height was 63.6 cm (50^th^ percentile), her weight was 5.8 kg (10^th^ percentile), and her head circumference was 48.5 cm (25^th^ percentile). Based on these clinical manifestations, the patient was initially suspected to have either Ohdo or Hallermann-Streiff syndrome. However, we discovered a novel heterozygous *DYRK1A* variant (c.1185dup, p.E396ter, [NM_001396.3]) on exon 8 by exome sequencing. The novel variant was only detected in the patient sample and was not present in the control population (variant databases from 1000 Genomes Project, ESP 6500, and ExAC). We confirmed the variant by Sanger sequencing (Fig. 1a). Parental segregation of the variant was negative, indicating that the mutation occurred in a sporadic form. Moreover, the patient’s brain MRI exhibited mild brain atrophy of both frontal lobes, thinning of the brainstem, hypoplasticity of the pituitary stalk and corpus callosum, and subcortical white matter hypomyelination, consistent with previously reported features of *DYRK1A* haploinsufficiency syndrome (Supplementary Fig. S1)^21^. After discovering that the patient’s disorder may be related to DYRK1A, rehabilitation therapy was initiated. At her 8-month follow-up (age: 13 months), the patient demonstrated delayed global developmental milestones and growth parameters. At 21 months of age, the patient developed seizures with generalized stiffness.

### Analysis of the structure and molecular dynamics of the DYRK1A-E396ter protein

**Figure 2:**
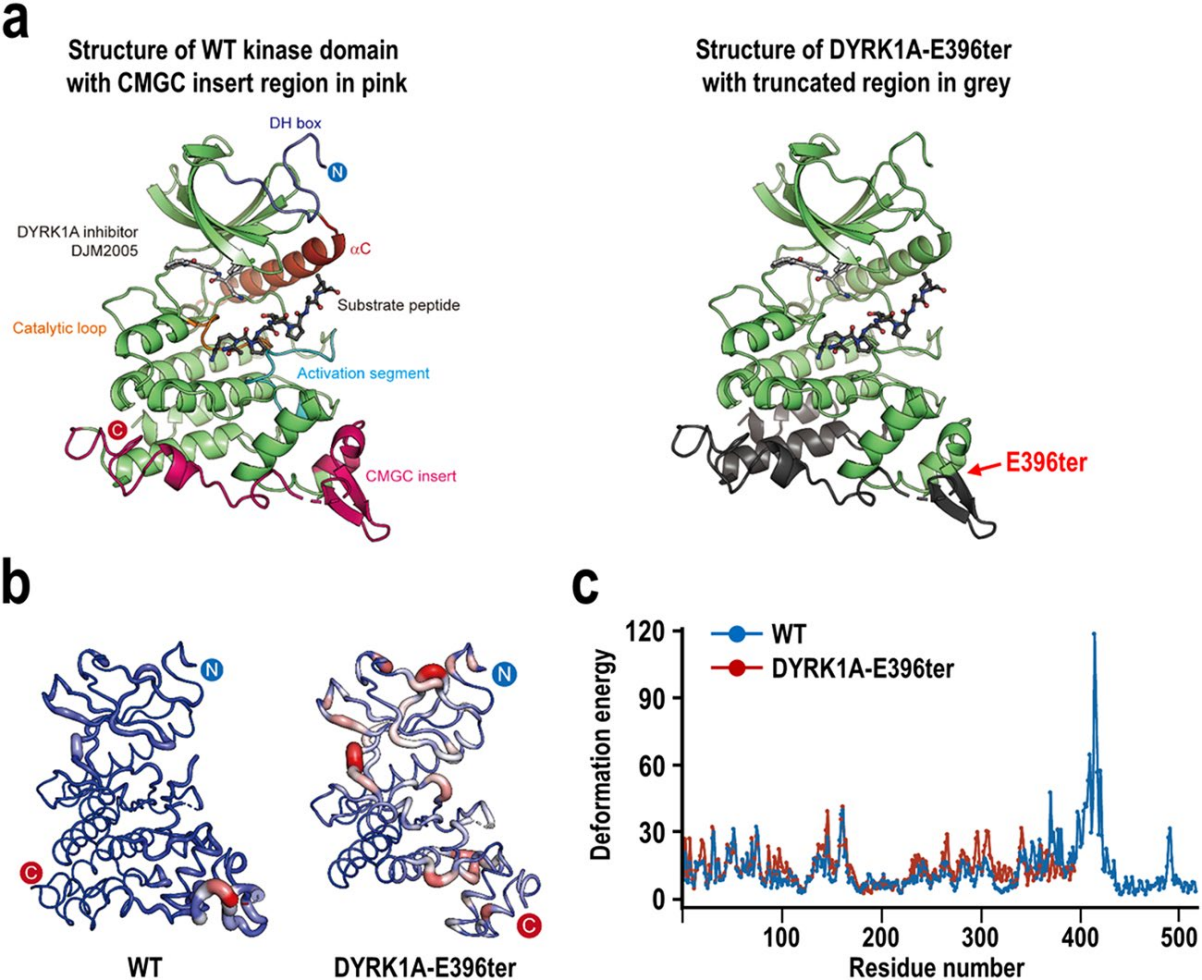
Analysis of the structure and molecular dynamics of the DYRK1A-E396ter protein. (**a**) Crystal structure of the human DYRK1A kinase domain in complex with its inhibitor, DJM2005 (PDB ID: 4MQ2) and substrate peptide is shown on the left. The C-helix, activation segment, catalytic loop, and CMGC insert are coloured in red, blue, orange, and hot pink, respectively. The N- and C-termini of the kinase domain of DYRK1A are represented by blue and red circles, respectively. The substrate peptide and inhibitor, DJM2005, are represented by a ball and stick model and coloured in dark and light grey, respectively. The predicted structure of DYRK1A-E396ter in complex with DJM2005 is shown on the right. The peptide sequences, which were not expressed in the mutant, are coloured dark grey and the location of the mutation (E396ter) is indicated by a red arrow. (**b**) Energy minimization of the wild-type and E396ter DYRK1A proteins was performed by using the MODELLER software, and the local molecular dynamics were assessed by using the Dynamut webserver with normal mode analysis function. Resulting molecular dynamics of the wild-type and E396ter DYRK1A proteins are presented in a tube style, which was generated by using PyMol software (version 1.3). The deformation energy is represented by thin to thick tubes coloured in blue (low), white (moderate), and red (high). (**c**) The deformation energy of the wild-type and E396ter DYRK1A proteins are coloured in blue and red, respectively.

The novel *DYRK1A* variant (c.1185dup) identified in this study was generated by the insertion of a single T at nucleotide position 1,185 of NM_001396.3. This insertion altered the translational frame, generated a premature translation termination codon (TGAG) at the E396 (GAG) position, and produced a truncated protein of 395 amino acids (DYRK1A-E396ter) (Fig. 1b). To gain a functional insight into the DYRK1A-E396ter protein, we primarily predicted its structure based on the crystal structure of the kinase domain in complex with its ATP-competitive inhibitor, DJM2005^32^ (Protein Data Bank ID: 4MQ2) and substrate peptide (Fig. 2a). Structurally, the nonsense mutation occurred in the β-sheet of the CMGC insert, which only exists in CMGC kinases, thereby producing a protein lacking most of CMGC insert, α-helix H, and α-helix I at the C-terminal end of the catalytic domain. For the analysis of the molecular dynamics, we next performed energy minimization of the truncated structure by using the MODELLER software, and then the local molecular dynamics of the wild-type and E396ter DYRK1A proteins were assessed by using the Dynamut webserver with normal mode analysis function (Fig. 2b). As a result, we found that the DYRK1A-E396ter protein had elevated deformation energy throughout the protein compared to the wild-type one. Relatively high deformation energy was shown in a catalytic loop, an activation segment, and the loop between α8 and α9, suggesting a potential reduction of protein stability (Fig. 2c).

### DYRK1A-E396ter is efficiently degraded by the proteasome

**Figure 3:**
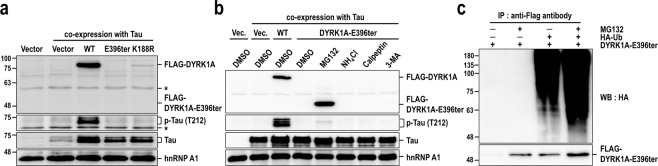
DYRK1A-E396ter is degraded by the ubiquitin-mediated proteasomal pathway and is intrinsically inactive. (**a**) FLAG-tagged DYRK1A-WT, DYRK1A-E396ter, and DYRK1A-K188R plasmids were transiently co-expressed with Tau protein in 293 T cells for 24 h. Total cell extracts were harvested and subjected to western blotting with anti-FLAG, anti-phosphorylated-Tau (at T212, p-Tau), and anti-Tau antibodies. HnRNP A1 served as a loading control. Asterisks indicate nonspecific proteins. (**b**) FLAG-tagged DYRK1A-WT and DYRK1A-E396ter were co-expressed with Tau protein and then treated with selective proteolytic pathway inhibitors: MG132 for the proteasome, NH_4_Cl for lysosome, calpeptin for calpain, and 3-methyladenine for autophagy. (**c**) FLAG-DYRK1A-E396ter and HA-ubiquitin were co-expressed for 24 h prior to MG132 treatment (10 μM, 11 h). Total cell extracts were harvested, FLAG-DYRK1A-E396ter was immunoprecipitated with anti-FLAG antibody, and polyubiquitination was detected by western blotting with an anti-HA antibody. Uncropped full-sized blots are presented in Supplementary Fig. S4.

To experimentally examine the protein stability of DYRK1A-E396ter in mammalian cells, we constructed a FLAG-tagged wild-type DYRK1A (FLAG-DYRK1A) expression clone and then introduced the mutation identified in the patient to produce a truncated protein of 395 amino acids (FLAG-DYRK1A-E396ter). In contrast to FLAG-DYRK1A-WT protein, FLAG-DYRK1A-E396ter protein was barely detectable by western blotting with an anti-FLAG antibody when overexpressed in 293 T cells (Fig. 3a). Since quantitative RT-PCR demonstrated that the transcripts were expressed at similar levels (Supplementary Fig. S2), we then examined the involvement of protein degradation. Protein degradation occurs in a specific and coordinated manner through major proteolytic systems, such as the proteasome, lysosome, calpains, and autophagy. To determine which systems contributed to the degradation of DYRK1A-E396ter, 293T cells expressing FLAG-DYRK1A-E396ter were treated separately with four selective inhibitors of each proteolytic degradation pathway. MG132, NH_4_Cl, calpeptin, and 3-methyladenine were used to inhibit proteasome-, lysosome-, calpain-, and autophagy-mediated protein degradation pathways, respectively. Western blotting with an anti-FLAG antibody revealed a prominent FLAG-DYRK1A-E396ter protein (~45 kDa) band only after treatment with MG132 (Fig. 3b) and not after treatment with any of the other inhibitors, suggesting that FLAG-DYRK1A-E396ter was degraded by the proteasome. Most proteins degraded by the proteasome are marked with (poly)ubiquitin chains. Thus, we analysed the ubiquitination of DYRK1A-E396ter by co-expressing FLAG-DYRK1A-E396ter and HA-ubiquitin in 293 T cells. Polyubiquitination of FLAG-DYRK1A-E396ter was observed at high levels when co-expressed with HA-ubiquitin and at even higher levels after additional treatment with MG132 (Fig. 3c). Together, these results indicate that DYRK1A-E396ter is degraded by the ubiquitin-dependent proteasomal degradation pathway.

### Loss-of-function of the DYRK1A patient mutant due to intrinsic inactivity

As the DYRK1A-E396ter protein was truncated at the catalytic domain, we sought to examine its kinase activity. We selected the Tau, a microtubule-associated protein, for the evaluation, because it is one of the most well-studied substrates of DYRK1A, and aberrant Tau phosphorylation is associated with the formation of neurofibrillary tangles in DS and Alzheimer’s disease^10,33^. Phosphorylation of Tau at the T212 residue is highly dependent on DYRK1A and was chosen as a marker of DYRK1A kinase activity^34^. Tau was transiently co-expressed with each FLAG-DYRK1A-WT, FLAG-DYRK1A-E396ter, and FLAG-DYRK1A-K188R^1^ (a catalytically inactive mutant of human DYRK1A) in 293 T cells. Phosphorylation of Tau at T212 was detected by western blotting with a phosphorylation-specific antibody. As previously reported, the co-expression of Tau and wild-type DYRK1A induced remarkable phosphorylation of Tau (Fig. 3a)^27^. In contrast, the DYRK1A-E396ter protein had little effect on Tau phosphorylation, which was similar to the DYRK1A-K188R protein.

We went on to examine whether FLAG-DYRK1A-E396ter possessed any intrinsic kinase activity when stabilized in the presence of MG132 (Fig. 3b). Despite a dramatic increase of FLAG-DYRK1A-E396ter protein level following the inhibition of proteasomal degradation, Tau phosphorylation was not rescued to an appreciable degree. These results indicate that the DYRK1A-E396ter mutant is catalytically inactive due to the truncation of the C-terminal region of the kinase domain. Additionally, we suggest that the intrinsic inactivity of DYRK1A-E396ter is the direct cause of its loss-of-function.

### DYRK1A-E396ter does not have a dominant-negative effect on wild-type DYRK1A

**Figure 4:**
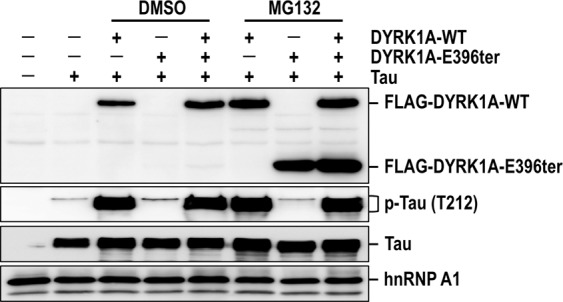
Effect of DYRK1A-E396ter on wild-type DYRK1A-induced Tau phosphorylation. DYRK1A-WT and DYRK1A-E396ter plasmids, alone or in a 1:1 combination, were co-transfected with the Tau plasmid for 24 h and then treated with MG132 (10 μM, 15 h). The subsequent procedure was identical to that described in the legend of Fig. 3a. Uncropped full-sized blots are presented in Supplementary Fig. S4.

Because both wild-type and mutant DYRK1A alleles are present in the patient’s cells, we investigated a potential dominant-negative effect of DYRK1A-E396ter on wild-type DYRK1A. The wild-type DYRK1A and DYRK1A-E396ter plasmids, alone or in a 1:1 combination, were co-transfected with the Tau plasmid, and the transfected cells were treated with MG132. As previously shown (Fig. 3a), Tau phosphorylation was strongly induced by the expression of wild-type DYRK1A. However, the additional co-expression of DYRK1A-E396ter did not impact Tau phosphorylation (Fig. 4). Similar results were observed even when the DYRK1A-E396ter protein was stabilized by MG132 treatment. Together, our findings indicate that DYRK1A-E396ter has little negative effect on wild-type DYRK1A with respect to Tau phosphorylation.

### Loss-of-function of the DYRK1A patient mutant in a *Drosophila* model

Because basic biological and neurological properties are highly conserved between human and *Drosophila*, *Drosophila* models are widely used to understand the molecular pathology of human diseases. Indeed, the *Drosophila* genome is nearly 75% homologous with human disease genes^35^, and the *Drosophila* minibrain (*mnb*) gene is 82% identical to its human homolog, *DYRK1A*^36,37^. Loss-of-function mutations in *mnb* result in reduced brain size with abnormal visual and olfactory behaviour due to defects in neurogenesis and brain development^38^, which recapitulates the microcephaly phenotype of *DYRK1A* haploinsufficiency in human patients. In addition, the tissue-specific overexpression of *mnb* also induces various phenotypic and neurological defects in central nervous system structure, consistent with various phenotypes of DS patients^39^.

**Figure 5:**
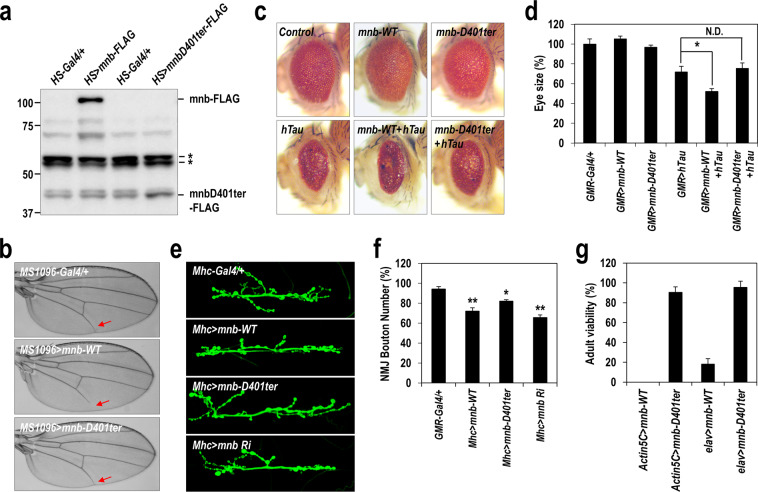
Loss-of-function of the *mnb-D401ter* mutant, the *Drosophila* equivalent of human DYRK1A-E396ter. (**a**) Transgenic flies ubiquitously overexpressing wild-type *mnb* or patient-derived truncated *mnb* (*mnb-D401ter)* were generated, and the expression of each mnb protein was analysed by western blotting with an anti-FLAG antibody. Asterisks indicate nonspecific proteins. Uncropped full-sized blots are presented in Supplementary Fig. S4. (**b**) Each mnb protein was specifically overexpressed in wing tissue using the *MS1096-gal* driver, and the resulting defects in L5 vein formation were analysed in adult flies. (**c**) Each mnb protein and/or human Tau was specifically overexpressed in the eye using the *GMR-gal* driver, and the resulting eye defects were analysed in adult flies. (**d**) Retinal surface areas were measured, and average eye sizes are presented as a percentage of the control. (**e**) Each mnb protein was overexpressed in muscle tissue using the *Mhc-gal* driver, and the resulting morphology of neuromuscular junctions in larval tissues were examined by visualization of the synaptic bouton. (**f**) The number of synaptic boutons was quantified and presented as a percentage of the control. (**g**) Each mnb protein was ubiquitously or neuro-specifically overexpressed using the *Actin5-* or *elav-gal* driver, respectively, and embryonic lethality was examined. Viability was presented as a percentage. Two-tailed Student’s *t-*tests were used to calculate *P-*values, which are depicted with an asterisk.

To evaluate the functionality of the DYRK1A-E396ter protein *in vivo*, we generated transgenic *Drosophila* harbouring wild-type *mnb* (*UAS-mnb-WT*) or truncated *mnb* (*UAS-mnb-D401ter*). Based on sequence comparison, *Drosophila* D401 was equivalent to human E396 (Supplementary Fig. S3). For tissue-specific overexpression, transgenic *mnb* flies were crossed with flies expressing tissue-specific *Gal4* drivers. The resulting expression of mnb-WT and mnb-D401ter proteins was analysed by western blotting of total protein extracts from transgenic flies (*HS-Gal4* > *UAS-mnb-WT* or *-D401ter*) (Fig. 5a). Similar to our results in mammalian cells (Fig. 3a), the D401ter protein was expressed at much lower levels than the wild-type protein.

Next, the function of the D401ter protein was examined by comparing the phenotypes of *mnb-D401ter* flies with those of *mnb-WT* flies. Overexpression of *mnb-WT* in wing tissue using the *MS1096-Gal4* driver showed shortening of the L5 vein in the adult wing, which we previously identified as one of the most prominent phenotypic defects^27^. In contrast, there was no recognizable phenotypic defect when the D401ter protein was overexpressed in the wing (Fig. 5b). Overexpression of human Tau in *Drosophila* is known to cause a severe eye degeneration phenotype^40^, which was worsened by co-expression with *mnb-WT* (Fig. 5c,d). Exacerbation of the eye degeneration phenotype is likely due to Tau hyperphosphorylation. In contrast, co-expression with *mnb-D401ter* had no additional effect.

As neuromuscular junction (NMJ) morphology of *Drosophila* larvae is used as a tool to assess neuronal synapse formation and integrity^41^, we analysed the effect of the D401ter mutation of *mnb* on NMJ morphology in transgenic flies. Overexpression of functional *mnb-WT* in the postsynaptic muscle tissue using *mhc-Gal4* significantly reduced the number of NMJ synaptic boutons. A similar effect was observed by siRNA-mediated knockdown of endogenous *mnb* (Fig. 5e,f). Compared with these results, *mnb-D401ter* overexpression had minimal effect on the formation of the NMJ bouton. Moreover, ubiquitous (*Actin5C-Gal4* > *UAS-mnb-WT*) or pan-neuronal (*elav-Gal4* > *UAS-mnb-WT*) expression of *mnb-WT* caused severe embryonic lethality of 80–100%, whereas *mnb-D401ter* expression barely affected fly viability or any other phenotypes (Fig. 5g). Collectively, these results indicate that the *mnb-D401ter* mutation in *Drosophila*, which corresponds to the human DYRK1A-E396ter mutation, results in loss of kinase activity and function.

## Discussion

In this study, we presented a patient with multiple congenital anomalies, including facial dysmorphism, developmental delay, and abnormalities in the cardiovascular system and brain structure (Supplementary Fig. S1). Exome sequencing and segregation analyses revealed that these phenotypic manifestations may be explained by a novel *de novo* heterozygous mutation of the *DYRK1A* gene. This novel mutation generated a translational termination codon and produced a C-terminally truncated protein (DYRK1A-E396ter) (Fig. 1a). Structurally, the DYRK1A-E396ter protein lacked the C-terminal end of the kinase domain, including the CMGC insert, α-helix H, and α-helix I. These regions, especially α-helices H and I, have been previously demonstrated to be required for the catalytic activity of DYRK1A. For instance, Arranz *et al*. showed that an R467ter DYRK1A mutant is completely inactive, and an F478 DYRK1A frame-shift mutant has less than 15% of the activity of wild-type DYRK1A. Their results indicate that even α-helix I, which is located at the C-terminal end of the kinase domain, is critically required for full kinase activity^42^. Consistent with this observation, we demonstrated that the DYRK1A-E396ter mutant was catalytically inactive. As this mutant protein was efficiently degraded by the proteasome and was barely detectable in mammalian cells, we evaluated its intrinsic kinase activity by inhibiting protein degradation with MG132. We found that even after protein stabilization, the DYRK1A-E396ter protein had little Tau-phosphorylating activity (Fig. 3b). According to an extensive analysis of DYRK1A missense mutants, lack of substrate phosphorylation activity is highly correlated with lack of tyrosine autophosphorylation^42^, which may be the case for the DYRK1A-E396ter protein.

More importantly, we further revealed that the mutant protein did not have a dominant-negative effect on the wild-type protein. Tau phosphorylation induced by the expression of the wild-type protein was barely affected by the additional expression of the mutant protein, even though the level of the mutant protein was recovered to the level of the wild-type protein by treatment with MG132 (Fig. 4). In the case of the two alleles are expressed in the patient’s heterozygous cells, this result indicates a lack of dominant-negative effect of the mutant protein, which would be the result of the combined contribution of two effects: reduced stability of the mutant protein and no interference of the mutant protein on the activities of the wild-type one. We further confirmed the lack of dominant-negative function of the mutant protein in *Drosophila* as an *in vivo* model. Transgenic *mnb-D401ter* flies, which have the *Drosophila* mutation equivalent to human DYRK1A-E396ter, had no recognizable phenotypic defects in the wings, eyes, and NMJs, and no severe embryonic lethality, whereas *mnb-WT* transgenic flies had severe phenotypic defects and lethality (Fig. 5). Collectively, these results clearly show that the DYRK1A-E396ter protein is not only catalytically inactive but is also completely non-functional in mammalian cells and fly models.

According to previous reports, the C-terminal end of the DYRK1A kinase domain is required for  protein stability as well as kinase activity^42–44^. Thus, loss of this region in the DYRK1A-E396ter mutant may directly affect protein stability. Indeed, we observed that the mutant protein was efficiently degraded by the ubiquitin-mediated proteasomal pathway and was consequently undetected in mammalian cells (Fig. 3b,c). It has been previously reported that DYRK1A degradation is mediated by binding of the E3 ubiquitin ligase SCF^βTrCP^ to the N-terminus of DYRK1A^45^. In addition, the CDC37/HSP90 chaperone has been suggested to regulate DYRK1A protein stability through interaction with its N-terminal lobe^46^. Whether these two mechanisms are associated with the degradation of the DYRK1A-E396ter protein needs further experimental validation.

In conclusion, we have identified a novel *de novo* DYRK1A nonsense mutation in a patient with *DYRK1A* haploinsufficiency syndrome. The mutation generates a C-terminally truncated protein at the β-sheet of the CMGC insert within the kinase domain that behaves as a loss-of-function mutant in both mammalian cell and *Drosophila* models.

## Supplementary information

Supplementary Information.
