# Supplementary material for: A novel de novo heterozygous DYRK1A mutation causes complete loss of DYRK1A function and developmental delay
Source: Sci Rep. 2020 Jun 17;10:9849. doi: 10.1038/s41598-020-66750-y (PMC7299959; doi:10.1038/s41598-020-66750-y)

**SUPPLEMENTARY INFORMATION**

**A novel *de novo* heterozygous *DYRK1A* mutation causes complete loss of DYRK1A function and developmental delay**

**Kyu-Sun Lee^1,2,#^, Miri Choi^3,4,#^, Dae-Woo Kwon^1,2,#^, Doyoun Kim^5^, Jong-Moon Choi^6^, Ae-Kyeong Kim^1^, Youngwook Ham^3,4^, Sang-Bae Han^4^, Sungchan Cho^3,7,*^, Chong Kun Cheon^8,9,*^**

^1^Bionanotechnology Research Center, Korea Research Institute of Bioscience and Biotechnology, 125 Gwahak-ro, Yuseong-gu, Daejeon 34141, Republic of Korea

^2^Department of Functional Genomics, KRIBB School of Bioscience, Korea University of Science and Technology, 217 Gajeong-ro, Gajeong-dong, Yuseong-gu, Daejeon 34113, Republic of Korea

^3^Natural Medicine Research Center, Korea Research Institute of Bioscience and Biotechnology, 30 Yeongudanji-ro, Ochang-eup, Cheongwon-gu, Cheongju-si, Chungbuk 28116, Republic of Korea

^4^College of Pharmacy, Chungbuk National University, 30-1 Yeonje-ri, Osong-eup, Heungduk-gu, Cheongju-si, Chungbuk 28644, Republic of Korea

^5^Innovative Target Research Center, Korea Research Institute of Chemical Technology, 141 Gajeong-ro, Jang-dong, Yuseong-gu, Daejeon 34114, Republic of Korea

^6^Green Cross Genome, Green Cross Laboratories, 107 Ihyeon-ro 30 beon-gil, Giheung-gu, Yongin-si, Gyeonggi 16924, Republic of Korea

^7^Department of Biomolecular Science, KRIBB School of Bioscience, Korea University of Science and Technology, 217 Gajeong-ro, Gajeong-dong, Yuseong-gu, Daejeon 34113, Republic of Korea

^8^Division of Medical Genetics and Metabolism, Department of Pediatrics, Pusan National University Children’s Hospital, Pusan National University School of Medicine, 20 Geumo-ro, Mulgeum-eup, Yangsan-si, Gyeongnam 50612, Republic of Korea

^9^Research Institute for Convergence of Biomedical Science and Technology, Pusan National University Yangsan Hospital, 20 Geumo-ro, Mulgeum-eup, Yangsan-si, Gyeongnam 50612, Republic of Korea

**Figure S1.** Phenotypic characteristics in the patient and negative parental segregation of the disorder. (a) Pedigree structure and segregation analysis of mutation in the family. The patient with the E396ter mutation in the *DYRK1A* gene is the third child of healthy Korean parents. The solid black circle represents the patient with the *DYRK1A* mutation. Only participants in the study whose DNA is available for analyses are numbered. (b) Facial features of the patient. Note the epicanthal fold, tented mouth, short and deep philtrum, deep-set eyes, bi-temporal narrowing, micrognathic face, wide nasal bone, sparse scalp hair, and prominent ears with underdeveloped ear lobes. (c,d,e,f) Images of the brain magnetic resonance imaging. Note that thinning of the brainstem (c), subcortical white matter hypomyelination (d), mild brain atrophy at both frontal lobes (e), and hypoplastic pituitary stalk and thinning of the corpus callosum (f) are compatible with the DYRK1A-related intellectual disability syndrome. Each phenotype is indicated by a red arrow.

**Figure S2.** The quantity of DYRK1A-E396ter transcript in comparison with wild-type DYRK1A. The mRNA transcript levels of transiently expressed FLAG-tagged wild-type DYRK1A, DYRK1A-E396ter, and DYRK1A-K188R were analyzed by qRT-PCR. The quantity of mRNA from the FLAG-tagged wild-type DYRK1A-expressing sample was set to 1, and the relative quantities of mRNA were calculated and presented. Human β-actin mRNA served as an internal control. Averages and SDs were determined from two independent experiments. Two-tailed Student’s *t*-test was used for a statistical analysis. N.S. means ‵not significant’.

**Figure S3.** Alignment of the amino acid sequence of human DYRK1A and *Drosophila* mnb kinase domains. Identical amino acids between the two sequences are indicated by black boxes and similar amino acid by grey boxes. E396 in human DYRK1A corresponds to D401 in *Drosophila* mnb, which is indicated by a red arrow.

**Figure S4.** Uncropped blot images. (a,b,c,d,e) Uncropped full size images of blots shown in Fig. 3a,b,c, Fig. 4, and Fig. 5a. Red boxed areas indicate lanes which have been cropped for final figure. Membranes were cut because of economical reasons.


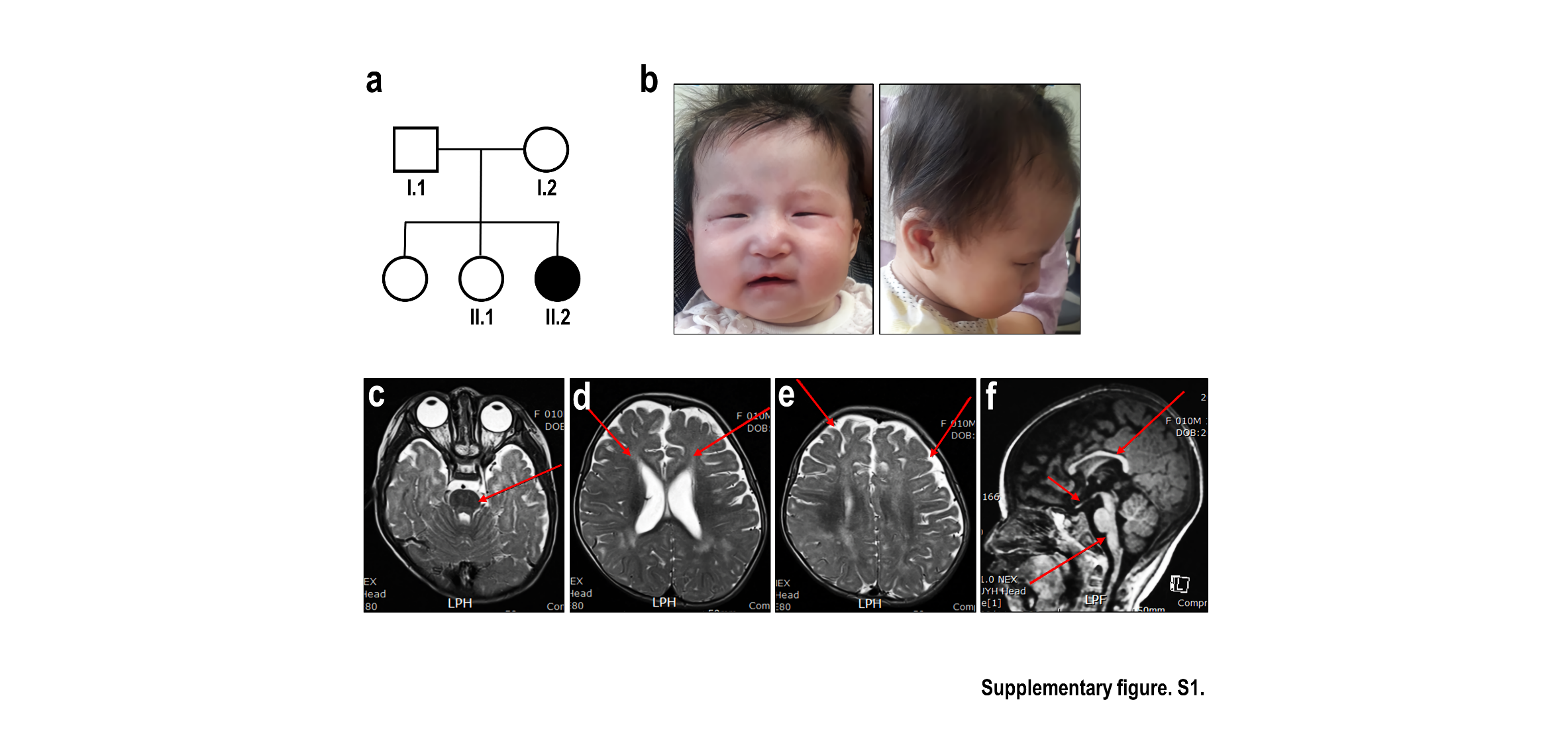


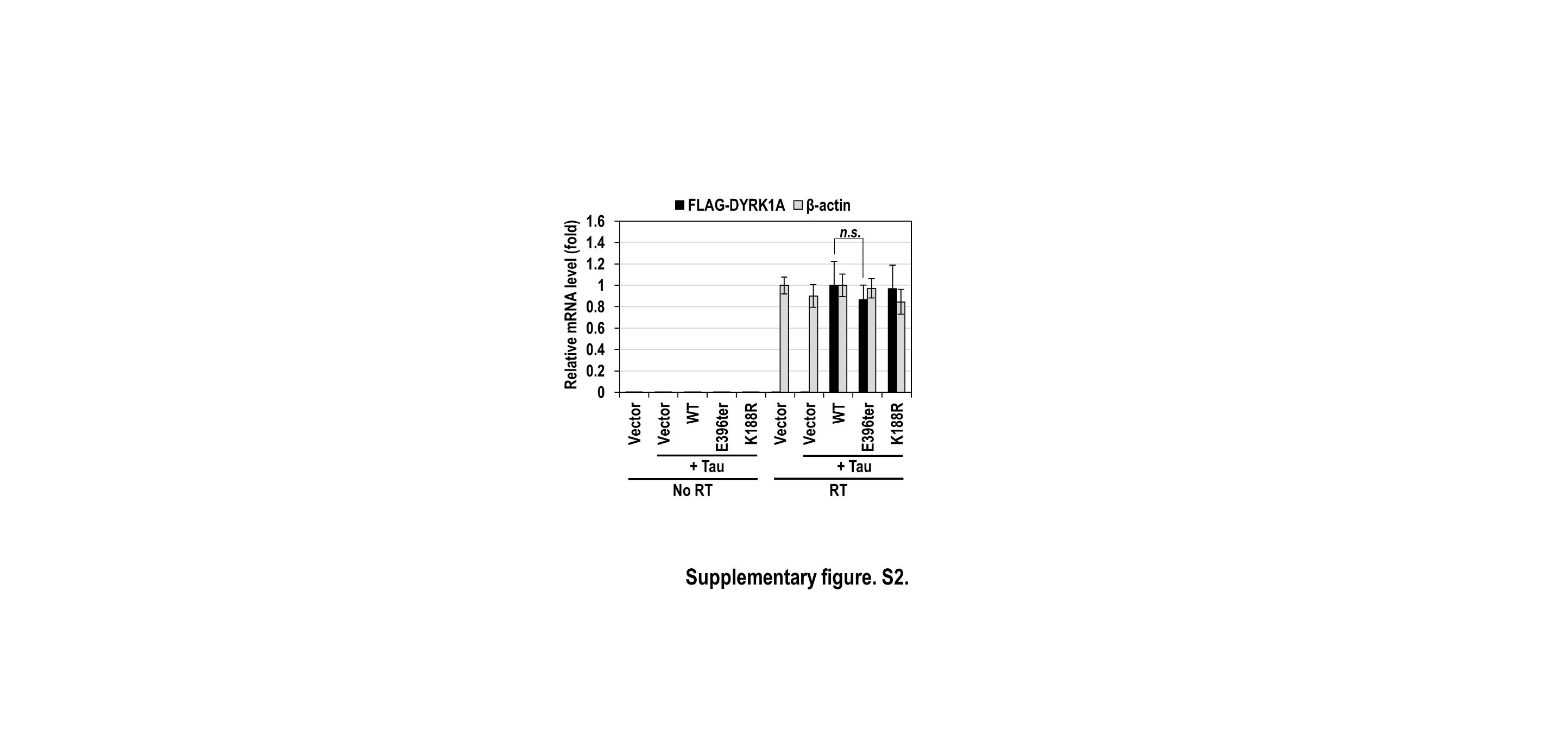


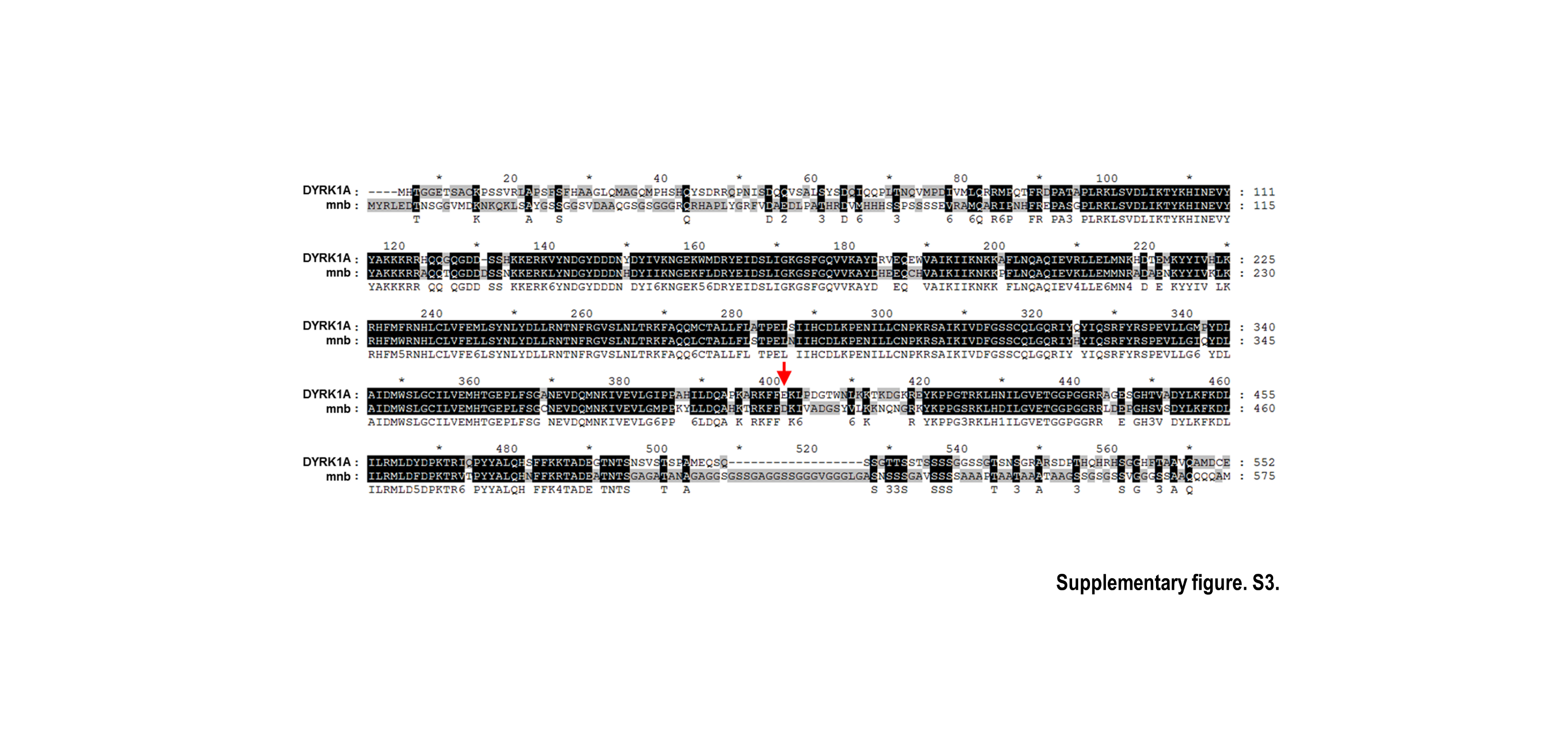


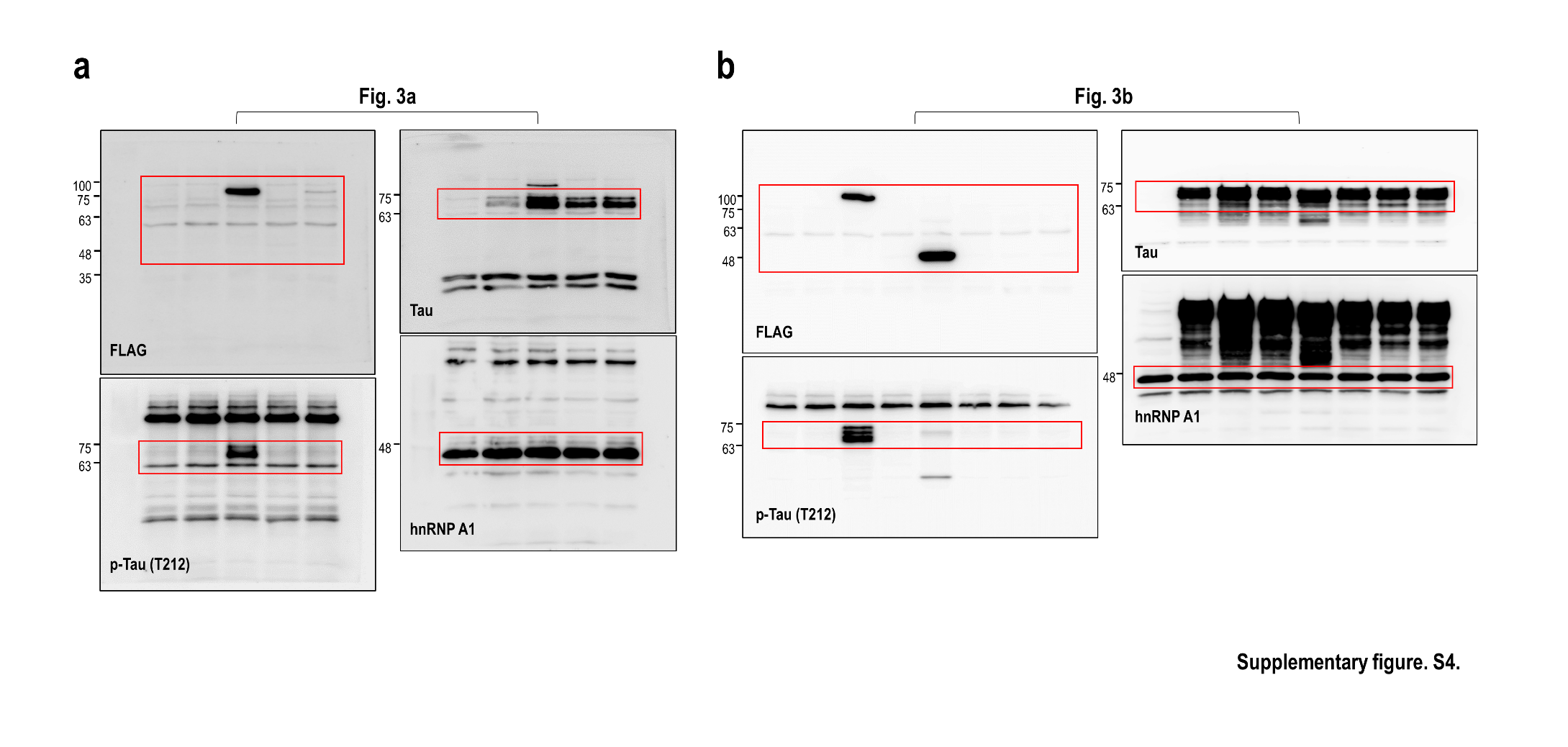


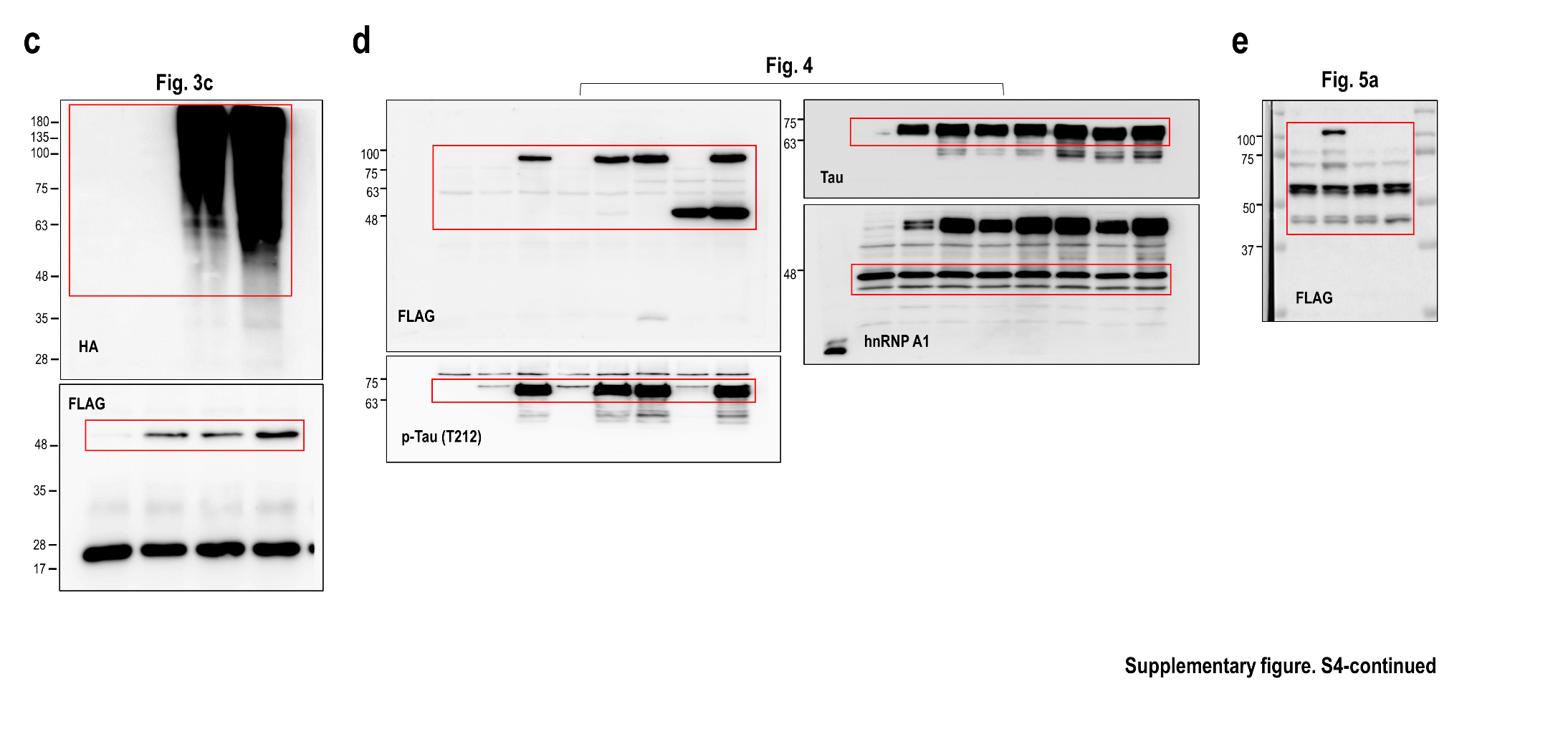

Supplement: Supplementary file 1 — Supplementary Information. [file 41598_2020_66750_MOESM1_ESM.docx]
